# Translation and cultural adaptation of the Reflux Finding Score into brazilian portuguese

**DOI:** 10.5935/1808-8694.20130009

**Published:** 2015-10-14

**Authors:** Andressa Guimarães do Prado Almeida, Taciane Brinca Soares Saliture, Álvaro Siqueira da Silva, Cláudia Alessandra Eckley

**Affiliations:** MD. Otorhinolaryngologist. MSc student - School scieces of Medical - Santa Casa de São Paulo; MSc student - School scieces of Medical - Santa Casa de São Paulo; MSc in tropical pathology - Federal University of the Amazonas; Preceptor of the Medical Residency Program in Otorhinolaryngology - Fundação Hospital Adriano Jorge/Universidade do Estado do Amazonas; PhD in Medicine - School of Medical Sciences - Santa Casa de São Paulo; Assistant Professor - Department of Otorhinolaryngology - Santa Casa de São Paulo. Santa Casa de São Paulo

**Keywords:** diagnosis, gastroesophageal reflux, laryngeal diseases, larynx, translations

## Abstract

The supraesophageal manifestations of Gastroesophageal Reflux Disease commonly known as Laryngopharyngeal Reflux (LPR) are highly prevalent. The diagnosis of LPR is challenging and mostly based on suggestive symptoms and signs of inflammation at the larynx and pharynx. In order to decrease the subjectivity of clinical assessment, a score based on endolaryngeal videolaryngoscopic findings, the Reflux Finding Score (RFS), was proposed by Belafasky et al. This score has proven to be highly sensitive and reproducible in the English language.

**Objective:**

Translate and culturally adapt the RFS into Brazilian Portuguese and test its reliability.

**Method:**

Following international guidelines, translation and back translation of the RFS was made by 2 independent professional translators who were native English speakers. The translated version of the RFS was then applied to the videolaryngoscopic images of 24 patients by 3 examiners twice with a 24-hour minimum interval between scoring sessions, and tested for intraobserver reliability.

**Results:**

The translation and cultural adaptation were carried out satisfactorily. Examiners applied the instrument, after brief technichal training, without difficulties. Intraobserver test re-test reliability and reproducibility were high.

**Conclusion:**

The Portuguese version of the RFS presents semantic similarity to the English version, and with reliability.

## INTRODUCTION

The gastroesophageal reflux disease (GERD) is one of the most common disorders of the digestive tract. It may be defined as a chronic diseases arising from a backflow of gastroduodenal content to the esophagus and/or adjacent organs, causing a varied spectrum of esophageal and/or extraesophageal signs and symptoms, associated or not with tissue damage^1^.

This is an disorder of high medical-social relevance, for it has a high and growing incidence, and for causing symptoms of varied intensity, which manifest for a long time. Supra-esophageal symptoms were reported by 58% of the patients who presented classical symptoms and, in these patients, there was a worsening in quality of life^2^.

One population study in Brazil, involving 22 large cities, in which they interviewed a sample of 13,959 individuals, they found 4.6% of the people assessed with pyrosis once a week and that 7.3% had such complaint twice or more times during the week^3^. Based on this data, it is estimated that the prevalence of classic GERD in our country is around 12%. There are esophageal and extraesophageal symptoms and the symptoms may or may not be followed by esophageal tissue lesions diagnosed by endoscopy.

Laryngopharyngeal reflux is a term being used since 1996 by Koufman to describe symptoms, tissue lesions or signs arising from the backflow of gastroduodenal content all the way to the upper aerodigestive tract^4^. Its frequent symptoms are: throat ache, globus pharyngeus, hawking, dysphonia, dry cough, laryngospasm fits^5^, together or not with symptoms such as heartburn, upper gastric pain, retrosternal pain^6^, ^7^, ^8^. Diagnostic evaluation includes laryngoscopic exam (videolaryngoscopy, nasal fibroscopy), in which it is possible to see some suggestive signs, however not specific, of reflux - such as diffuse laryngeal edema and hyperemia, vocal fold edema, vestibular fold edema, subglottic mucosa edema, interarytenoid region hypertrophy, thick endolaryngeal mucus and granuloma or granulation tissue^6^, ^7^, ^8^. There is also a correlation with subglottic stenosis and laryngeal carcinoma^9^, ^10^, ^11^, ^12^. Its diagnose is challenging, because these inflammatory signs are common to other diseases affecting the laryngopharyngeal segment.

Upper digestive tract endoscopy has low sensitivity to diagnose laryngopharyngeal reflux, and a large number of patients with this supraesophageal manifestation of GERD do not have esophagitis or any other sign suggesting hypotonia of the lower esophageal sphincter (LES).

Longstanding monitoring tests of the esophagus help diagnose the disease, although highly specific, they have low sensitivity, especially in cases of laryngopharyngeal reflux. Esophageal pH monitoring may be carried out with one or two sensors; and the second type is paramount when suspecting of supra-esophageal reflux^13^, ^14^, ^15^. The difficulties associated with test acceptance and its technical limitations (it only detects episodes of acid and liquid reflux) led to the development of esophageal impedance pH monitoring, which technique enables the assessment of liquid or gas, acid or non-acid reflux^16^. However, both exams are difficult to have access to, especially in developing countries, besides poor patient compliance because of the nuisance of it. Adding to this fact, there is no consensus on the normality parameters for these tests in patients with laryngopharyngeal reflux.

Thus, the diagnosis of laryngopharyngeal reflux is mostly based on the presence of symptoms and suggestive laryngoscopic signs. In order to minimize the test's subjectivity, especially those associated with image videolaryngoscopy tests, a group of researchers proposed a scoring system, the Reflux Finding Score, based on endolaryngeal inflammatory findings, presumably suggestive of involvement by reflux. Such index was validated for the English language in 2001 by Belafsky et al. and has been utilized in the world literature as a diagnostic parameter for laryngopharyngeal reflux. The scoring system assigns grades of intensity to inflammatory signs and the presence or absence of lesions suggesting the disease. The Reflux Finding Score has shown high reproducibility and reliability, and a patient with a score higher than 7 points has a 94% possibility of having laryngopharyngeal reflux^17^. The instrument has also been utilized to monitor disease progress and response to treatment.

Cultural diversity requires that for such instrument to be utilized in Brazil, it has to be translated according to guidelines which, if complied with, result in semantic, content and concept equivalence with the original model, for later validation.

The goal of this paper was to translate the Reflux Finding Score into Brazilian Portuguese, with proper cultural adaptation and test its reliability.

## METHOD

Following international guidelines^18^, ^19^, ^20^, we ran the following translation stages:

1. Translation into Portuguese, carried out independently by two Brazilian ENT physicians with experience in laryngology and proficiency in the English language;
2. Consensus between the two translators regarding a Portuguese version, together with another bilingual translator (co-author of this study);
3. Back-translation carried out independently by two native speakers of English;
4. Comparison of the back-translation with the original version vis-à-vis concept, semantics and content equivalence; translation done by the committee made up of the two translators, two translators for the retranslation and one of the coauthors of the study;
5. Reliability test of the pre-final version in Brazil, carried out by three otorhinolaryngologists, who will be the professionals to use the instrument.

Three trained otorhinolaryngologists carried out the reliability test by means of the analysis of 24 videolaryngoscopy exams from a database of a tertiary university hospital. They blindly applied the translated instrument (the tests referred to patients with or without laryngopharyngeal reflux, recorded in a random fashion). Each one of the three experienced examiners (A, B and C) employed the Endolaryngeal Reflux Findings Scale twice, with a minimum interval of 24 hours. The selected images were randomly mixed in two different sequences for the test and retest, to minimize bias.

The statistical analysis was carried out by percentage of agreement, using colors. The green color was used when the examiner scored the subdomain in an identical fashion in the two blind assessments. The yellow color was utilized for errors considered small/acceptable, in other words, if the examiner differed only in one grade in his/ her analysis - it was considered satisfactory, for instance, if in the first evaluation - test - the diffuse edema was scored as mild (1 point) and in the second - retest - as moderate (2 points), among five possible scores (0, 1,2,3,4 or 5); another example, if the examiner scored “4” in one assessment and “3” in the other, and so on and so forth. This was pertaining because it was a qualitative assessment, in which errors are more frequent when compared with quantitative assessments.

The red color was utilized for errors greater than one grade or scoring difference greater than 1. It is worth stressing that subdomains “1” (subglottic edema), “7” (granuloma/granulation tissue) and “8” (granuloma/ granulation) were considered as green for the “matching answers” and the red color for the errors, since they are classified only as 0 or 1 (absent or present).

The test and retest data from each examiner was plotted in an Excel 97-2003 spreadsheet and, then, exported to the EpiInfo software - which generated the tables for each subdomain and each examiner, comparing the test and retest coherence (reliability). This table was, once again, exported to excel with colors, and we calculated the percentage of “matching” results, acceptable errors and errors from the different examiners for each subdomain.

Besides the description of the errors in percentage values, acceptable errors and errors, we also calculated the Kappa index used to assess intraexaminer agreement for each subdomain.

## RESULTS

Based on the Reflux Finding Score diagnostic instrument (Table 1) in English (original language), the two translations were carried out in an independent way, both by otorhinolaryngologists with proficiency in English (Table 2, Table 3).

**Table 1:** Original instrument in English.

Reflux Finding Score (RFS)	
Subglottic edema	0 absent
	2 present
Ventricular obliteration	2 partial
	4 complete
Erythema/hyperemia	2 arytenoids only
	4 diffuse
Vocal fold edema	1 mild
	2 moderate
	3 severe
	4 polypoid
Diffuse laryngeal edema	1 mild
	2 moderate
	3 severe
	4 obstructing
Posterior commissure hypertrophy	1 mild
	2 moderate
	3 severe
	4 obstructing
Granuloma/granulation tissue	0 absent
	2 present
Thick endolaryngeal mucus	0 absent
	2 present

**Table 2:** Independent translation carried out by the first translator.

Escore de achados de refluxo	
Edema subglótico	0 ausente
	2 presente
Obliteração ventricular	2 parcial
	4 completa
Eritema/Hiperemia	2 somente em aritenoides
	4 difusa
Edema de pregas vocais	1 leve
	2 moderado
	3 grave
	4 polipoide
Edema laríngeo difuso	1 leve
	2 moderado
	3 grave
	4 obstrutivo
Hipertrofia da comissura posterior	1 leve
	2 moderado
	3 grave
	4 obstrutivo
Granuloma/Tecido de granulaçâo	0 ausente
	2 presente
Muco endolaríngeo espesso	0 ausente
	2 presente

**Table 3:** Independent translation carried out by the second translator.

Escala de achados de refluxo	
Edema subglótico	0 ausente
	2 presente
Obliteração dos ventrículos	2 parcial
	4 completa
Eritema/Hiperemia	2 somente das aritenoides
	4 difusa
Edema das pregas vocais	1 leve
	2 moderado
	3 grave
	4 polipoide
Edema laríngeo difuso	1 leve
	2 moderado
	3 grave
	4 obstrutivo
Hipertrofia da comissura posterior	1 leve
	2 moderado
	3 grave
	4 obstrutivo
Granuloma/Tecido de granulação	0 ausente
	2 presente
Muco endolaríngeo espesso	0 ausente
	2 presente

Both translations were analyzed by the two translators together with a third bilingual translator, coauthor of the study and, by means of a consensus, we then carried out a third translation (Table 4).

**Table 4:** Consensus between the two translators and one of the coauthors of this study.

Escala de achados de refluxo	
Edema subglótico	0 ausente
	2 presente
Obliteração dos ventrículos	2 parcial
	4 completa
Eritema/Hiperemia	2 somente das aritenoides
	4 difusa
Edema das pregas vocais	1 leve
	2 moderado
	3 grave
	4 polipoide
Edema laríngeo difuso	1 leve
	2 moderado
	3 grave
	4 obstrutivo
Hipertrofia da comissura posterior	1 leve
	2 moderado
	3 grave
	4 obstrutivo
Granuloma/Tecido de granulação	0 ausente
	2 presente
Muco endolaríngeo espesso	0 ausente
	2 presente

From that version, we did a retranslation by two North Americans/native speakers of English, independently. Both versions were similar (Table 5).

**Table 5:** Independent retranslation carried out by two North-American native speakers of English.

Reflux Finding Score (RFS)	
Subglottic edema	0 absent
	2 present
Ventricular obliteration	2 partial
	4 complete
Erythema/hyperemia	2 arytenoids only
	4 diffuse
Vocal fold edema	1 mild
	2 moderate
	3 severe
	4 polypoid
Diffuse laryngeal edema	1 mild
	2 moderate
	3 severe
	4 obstructing
Posterior commissure hypertrophy	1 mild
	2 moderate
	3 severe
	4 obstructing
Granuloma/granulation tissue	0 absent
	2 present
Thick endolaryngeal mucus	0 absent
	2 present

In a new committee, made up of the four translators, besides one of the study's co-author, we analyzed the concept, semantics and content equivalences with a satisfactory result, given the noted similarity between the original version and the retranslation. The committee chose to add the word “endolaryngeal” to the Reflux Findings Scale, for considering that it facilitates its understanding. Thus, the final name became: Endolaryngeal Reflux Findings Scale. Another change was the term: “posterior commissure”, replaced by “interarytenoid region” according to the nomina anatomica^21^, accepted by all the translators. The translation proposed by the authors into Brazilian Portuguese of the Reflux Finding Score can be found on Table 6.

**Table 6:** Final version of the Reflux Finding Score translation into Brazilian Portuguese.

Escala de achados endolaríngeos de refluxo	
Edema subglótico	0 ausente
	2 presente
Obliteração dos ventrículos	2 parcial
	4 completa
Eritema/Hiperemia	2 somente das aritenoides
	4 difusa
Edema das pregas vocais	1 leve
	2 moderado
	3 grave
	4 polipoide
Edema laríngeo difuso	1 leve
	2 moderado
	3 grave
	4 obstrutivo
Hipertrofia da região interaritenóidea	1 leve
	2 moderado
	3 grave
	4 obstrutivo
Granuloma/Tecido de granulação	0 ausente
	2 presente
Muco endolaríngeo espesso	0 ausente
	2 presente

After analyzing the 24 tests recorded on DVD, carried out by three examiners (A, B and C) blindly and with a minimum interval of 24 hours and the test and retest of the same exam, there were the following intraexaminer agreements (Graph 1, Graph 2, Graph 3, Graph 4, Graph 5, Graph 6, Graph 7, Graph 8).

**Graph 1:**
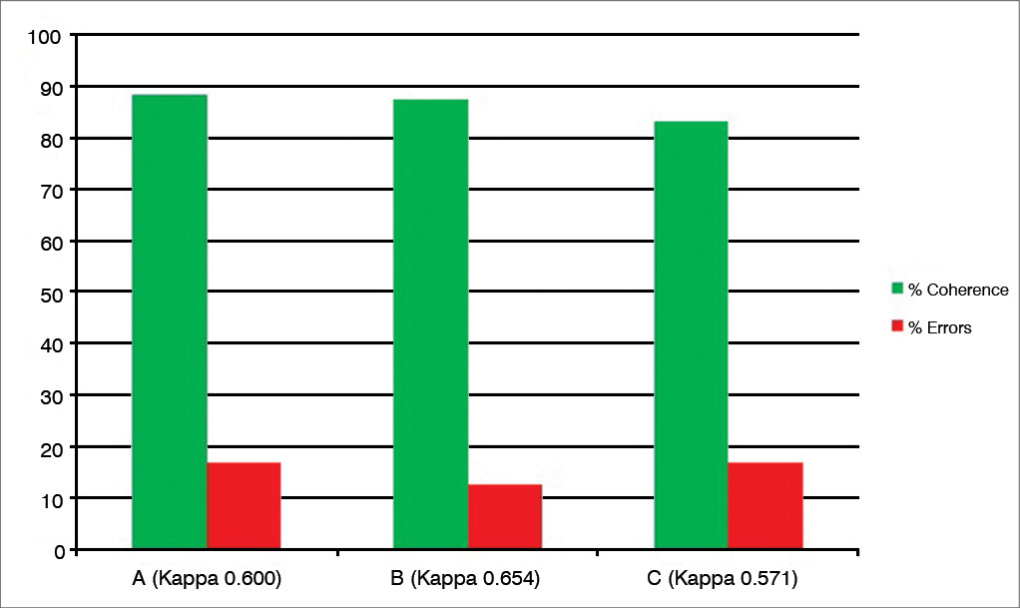
Percentage value of intraexaminer agreement (A, B and C) for the subglottic edema subdomain.

**Graph 2:**
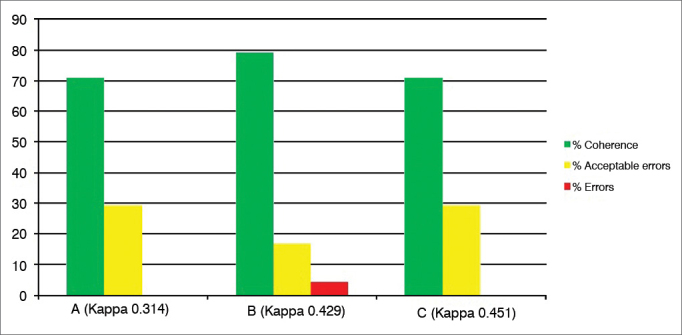
Percentage value of intraexaminer agreement (A, B and C) for the ventricle obliteration subdomain.

**Graph 3:**
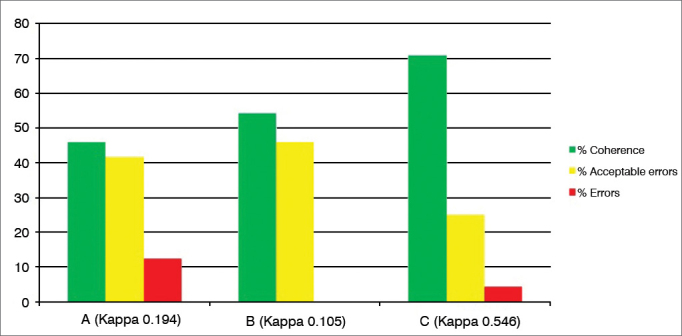
Percentage value of intraexaminer agreement (A, B and C) for the erythema/hyperemia subdomain.

**Graph 4:**
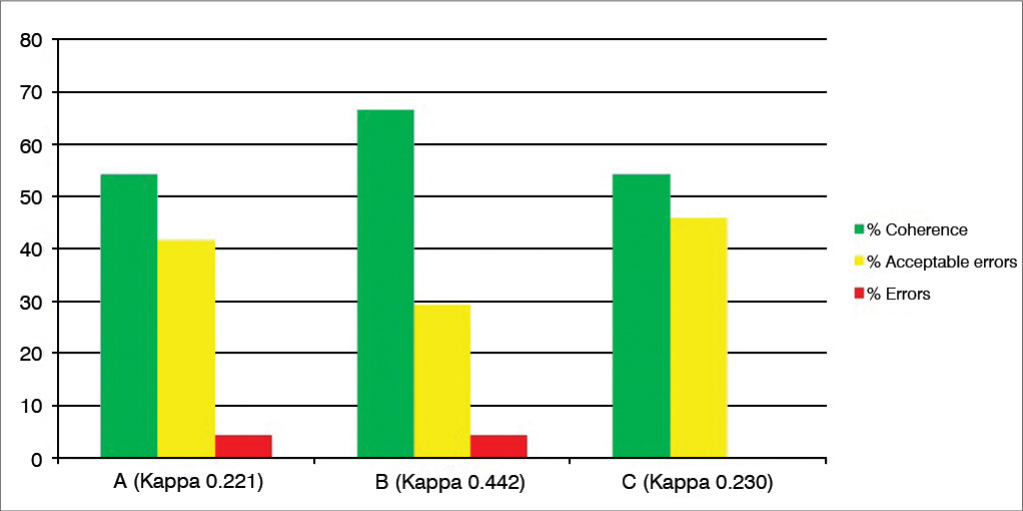
Percentage value of intraexaminer agreement (A, B and C) for the vocal fold edema subdomain.

**Graph 5:**
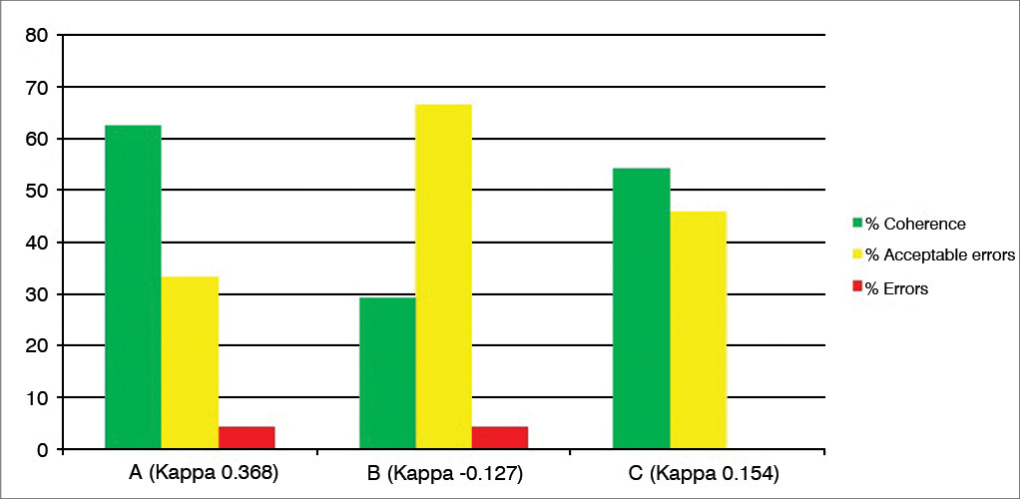
Percentage value of intraexaminer agreement (A, B and C) for the diffuse laryngeal edema subdomain.

**Graph 6:**
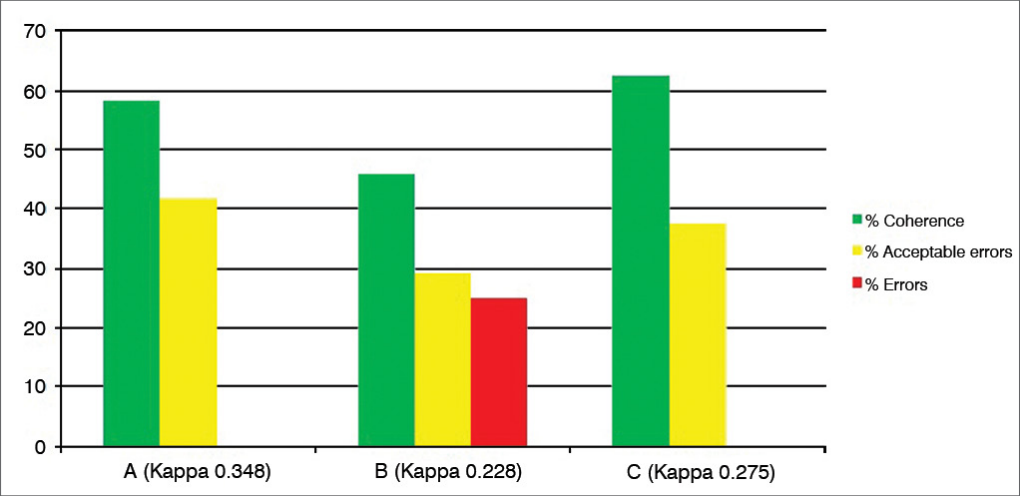
Percentage value of intraexaminer agreement (A, B and C) for the interarytenoid region hypertrophy subdomain.

**Graph 7:**
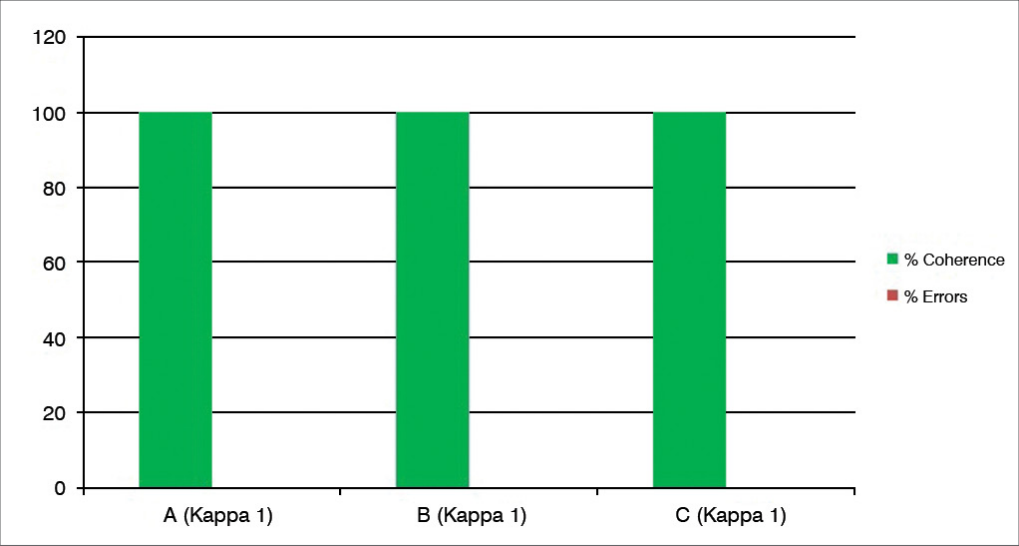
Percentage value of intraexaminer agreement (A, B and C) for the granuloma/granulation tissue subdomain.

**Graph 8:**
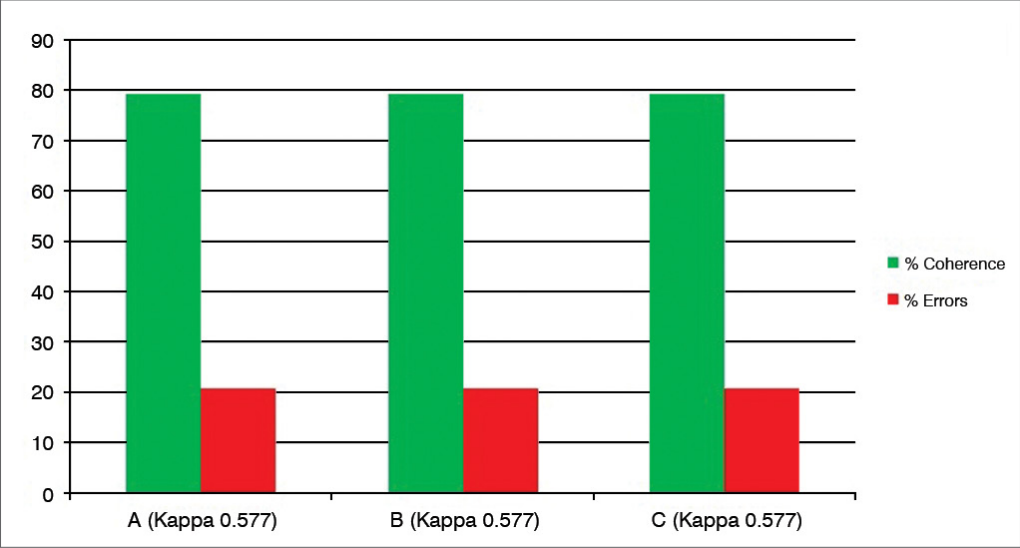
Percentage value of intraexaminer agreement (A, B and C) for the thick endolaryngeal mucus subdomain.

## DISCUSSION

Since this is a diagnostic instrument, to be employed by physicians with expertise in laryngology, there were no difficulties in understanding the technical terms and its content. Both the translation and the retranslation had 99% agreement in all the subdomains, and the minimum differences found (for instance, the translation of the title, which was sometimes translated as score, and sometimes as scale), were solved after consensus with expert physicians and translators, not involving disagreement as far as content is concerned. We chose to add to the instrument's name the term “endolaryngeal”, for better understanding and ease of clinical use. Another change in the Portuguese version was the translation of the “posterior commissure” for “interarytenoid region”, corresponding to the same anatomical region, in such a way that we followed the international nomina anatomica^21^.

After the initial training concerning the technical aspects of the scale, all the otorhinolaryngologists were capable of employing the instrument and did not report understanding difficulties as to the terms present in the subdomains.

We then employed the test and retest in 24 videolaryngoscopy exams, with the results expressed in the Graph 1, Graph 2, Graph 3, Graph 4, Graph 5, Graph 6, Graph 7, Graph 8, for the three examiners (A, B and C).

The “subglottic edema” (Graph 1) and “thick laryngeal mucus” (Graph 8) are the ones without the possibility of “acceptable errors”, in other words, the examiner assessing the exam in the first moment (test) and in the second moment (retest) should have the same scores so as not to consider “error”. In these two subdomains, the matching was 80% or more, and the errors were 20% or less, considered satisfactory as intelligibility of the question and intraobservers' reproducibility (the Kappa varied between 0.57 and 0.65) are concerned.

Other subdomains in which “acceptable errors” were not considered because there were only two options for answers were: “granuloma/granulation tissue” (Graph 7), in which there was 100% of intraobservers and interobserver coherence (Kappa 1).

The subdomain “Ventricular Obliteration” (Graph 2) presented 70-80% intra-observer reliability, 20-50% acceptable errors, and 5% of errors. Considering that this is a subjective measure (incomplete or complete obliteration), a satisfactory coherence was observed (Kappa 0.3 to 0.45). It is important to note that Kappa does not consider acceptable errors, thus the low Kappa scores with an error rate of only 5%.

For the third subdomain, “Erythema/Hyperemia” (Graph 3), considered one of the most subjective, there was 40% to 70% matching responses, “acceptable errors” 20% to 50% and “errors” 0% to 13, also considered satisfactory understanding of the subdomain and acceptable percentage of errors considering the method's subjectivity (Kappa 0.1 to 0.54).

Subdomains “Vocal fold edema” (Graph 4), “Diffuse laryngeal edema” (Graph 5) and “Interarytenoid region hypertrophy” (Graph 6) are the ones with the most variables (scoring possibilities between 0 and 5), besides a high degree of subjectivity (classify as mild, moderate, high, polypoid/obliterating). Despite these considerations, there was a percentage of “errors” lower than 5% in subdomains 4 and 5, “coherence” between 30% and 70% and “acceptable errors” between 30 and 66% (Kappa 0.12 to 0.36).

In subdomain 6 (“interarytenoid region hypertrophy”), two of the three examiners did not make any mistake comparing test and retest; however, there was a substantial difference in response from examiner B, who made an error percentage of 25% - considered non-satisfactory. The Kappa index did not show this difference, nonetheless, assessing the graph of percentages, it is easy to understand. We considered a greater difficulty in training the examiner for assessing this subdomain, and not a failure of the instrument, since the other two examiners had satisfactory intraobserver coherence (50% to 60% coherence and acceptable errors between 30 and 40%, Kappa of 0.27 and 0.34).

This is a tool to be utilized by laryngologists who already have experience and knowledge about laryngopharyngeal reflux, which will add objectiveness to the diagnosis, besides providing a parameter for following up the patients with this disorder. It is fast and easy to train the examiners, nonetheless, since it is subjective agreement variable with each subdomain, it is possible that some subdomain be difficult to understand. The examiners' response distribution was similar in all the subdomains, except the sixth: “interarytenoid region hypertrophy”, in which one examiner incurred in 25% error comparing test and retest (intraobserver), while the other two examiners had 0% of error, that is, in one subdomain, only one of the three examiners found it difficult to understand and deploy the instrument. It is worth stressing that to effectively use this diagnostic instrument, after translation, there is the final stage of method validation, which is still ongoing.

## CONCLUSION

The Endolaryngeal Reflux Findings Scale has concept, semantics and content similarities with the Reflux Finding Score, besides reliability.

## References

[1] Moraes-Filho J, Cecconello I, Gama-Rodrigues J, Castro L, Henry MA, Meneghelli UG (2002). Brazilian consensus on gastroesophageal reflux disease: proposals for assessment, classification, and management. Am J Gastroenterol..

[2] Mearin F, Ponce J, Ponce M, Balboa A, Gónzalez MA, Zapardiel J. (2012). Frequency and clinical implications of supraesophageal and dyspeptic symptoms in gastroesophageal reflux disease. Eur J Gastroenterol Hepatol..

[3] Moraes-Filho JP, Chinzon D, Eisig JN, Hashimoto CL, Zaterka S. (2005). Prevalence of heartburn and gastroesophageal reflux disease in the urban Brazilian population. Arq Gastroenterol..

[4] Koufman J, Sataloff RT, Toohill R. (1996). Laryngopharyngeal reflux: consensus conference report. J Voice..

[5] Bortolotti M. (1989). Laryngospasm and reflex central apnoea caused by aspiration of refluxed gastric content in adults. Gut..

[6] Bain WM, Harrington JW, Thomas LE, Schaefer SD. (1983). Head and neck manifestations of gastroesophageal reflux. Laryngoscope..

[7] Chodosh PL. (1977). Gastro-esophago-pharyngeal reflux. Laryngoscope..

[8] Koufman JA. (1991). The otolaryngologic manifestations of gastroesophageal reflux disease (GERD): a clinical investigation of 225 patients using ambulatory 24-hour pH monitoring and an experimental investigation of the role of acid and pepsin in the development of laryngeal injury. Laryngoscope..

[9] Little FB, Koufman JA, Kohut RI, Marshall RB. (1985). Effect of gastric acid on the pathogenesis of subglottic stenosis. Ann Otol Rhinol Laryngol..

[10] Jindal JR, Milbrath MM, Shaker R, Hogan WJ, Toohill RJ. (1994). Gastroesophageal reflux disease as a likely cause of “idiopathic” subglottic stenosis. Ann Otol Rhinol Laryngol..

[11] Morrison MD. (1988). Is chronic gastroesophageal reflux a causative factor in glottic carcinoma?. Otolaryngol Head Neck Surg..

[12] Ward PH, Hanson DG. (1988). Reflux as an etiological factor of carcinoma of the laryngopharynx. Laryngoscope..

[13] DeMeester TR, Wang CI, Wernly JA, Pellegrini CA, Little AG, Klements-chitsch P (1980). Technique, indications, and clinical use of 24 hour esophageal pH monitoring. J Thorac Cardiovasc Surg..

[14] Nasi A, de Moraes-Filho JPP, Cecconello I. (2006). Doença do refluxo gas-troesofágico: revisão ampliada. Arq Gastroenterol..

[15] Smit CF, Mathus-Vliegen LM, Devriese PP, Schouwenburg PF, Ku-pperman D. (2000). Diagnosis and consequences of gastropharyngeal reflux. Clin Otolaryngol Allied Sci..

[16] Pritchett JM, Aslam M, Slaughter JC, Ness RM, Garret CG, Vaezi MF. (2009). Efficacy of esophageal impedance/pH monitoring in patients with refractory gastroesophageal reflux disease, on and off therapy. Clin Gastroenterol Hepatol..

[17] Belafsky PC, Postma GN, Koufman JA. (2001). The validity and reliability of the reflux finding score (RFS). Laryngoscope..

[18] Beaton DE, Bombardier C, Guillemin F, Ferraz MB. (2000). Guidelines for the process of cross-cultural adaptation of self-report measures. Spine (Phila Pa 1976)..

[19] Ferraz MB. (1997). Cross cultural adaptation of questionnaires: what is it and when should it be performed?. J Rheumatol..

[20] Sousa VD, Rojjanasrirat W. (2011). Translation, adaptation and validation of instruments or scales for use in cross-cultural health care research: a clear and user-friendly guideline. J Eval Clin Pract..

[21] Sociedade Brasileira de Anatomia (2001). Terminologia Anatômica.

